# Positive effects of health behaviors acquired during the COVID-19 pandemic process on the prevention of other infectious diseases

**DOI:** 10.55730/1300-0144.5745

**Published:** 2023-10-12

**Authors:** Asiye Çiğdem ŞİMSEK, Turan BUZGAN, Fatma Nur BARAN AKSAKAL, Şuayip BİRİNCİ, Hülya ŞİRİN

**Affiliations:** 1 Department of Public Health, Gulhane School of Medicine, University of Health Sciences, Ankara, Turkiye; 2 Department of Infectious Diseases, Faculty of Medicine, Yildirim Beyazit University, Ankara, Turkiye; 3 Department of Public Health, Faculty of Medicine, Gazi University, Ankara, Turkiye; 4 Ministry of Health, Republic of Turkiye, Ankara, Turkiye

**Keywords:** COVID-19, hygiene, mask, distance, travel restrictions

## Abstract

**Background/aim:**

It was aimed to evaluate the positive effects of health behaviors (general hygiene, wearing face masks, physical distancing, and travel restrictions) acquired during the coronavirus disease 2019 (COVID-19) pandemic on the prevention of other infectious diseases in Ankara Province, Türkiye.

**Materials and methods:**

This study was designed retrospectively. Among the notifiable group A infectious diseases, acute intestinal infections (AIIs) with International Classification of Diseases, Tenth Revision diagnosis codes A09 (diarrhea and gastroenteritis presumed to be of infectious origin), R11 (nausea and vomiting), and K52 (other noninfectious gastroenteritis and colitis), as well as influenza, tuberculosis, measles, varicella, malaria, and meningococcal meningitis were included in the scope of this study.

The data of the selected infectious diseases in Ankara Province for the last 2 years before the pandemic (January 2018–December 2019) and for the 2-year period of the pandemic (January 2020–December 2021) were analyzed after checking the data. The number of cases were presented as frequencies, the 1-sample chi-squared test was used in the statistical analysis and the statistical significance level (α) was taken as 0.05.

**Results:**

The findings for each disease/disease group were discussed under separate headings. Comparing the prepandemic period (2018–2019) with the pandemic period (2020–2021), the decreases in the number of cases of selected infectious diseases, except influenza, were statistically significant.

**Conclusion:**

Undoubtedly, the experience gained from the pandemic struggle will guide us in shaping our future lives. From this point forward, we should be aware that living in crowded environments and as a highly mobile population, that unhygienic habits are unfavorable for the spread of all infectious diseases, and we should take care to continuously apply the precautions for healthy living in our new lifestyle.

## 1. Introduction

Infectious disease outbreaks have been one of the most important problems to have changed the course of human history. Millions of people have lost their lives or had to live with disease sequelae for a lifetime due to epidemics that sometimes lasted for many years. In the last 50 years, acquired immunodeficiency syndrome (AIDS), Ebola, severe acute respiratory syndrome (SARS), and Middle East respiratory syndrome (MERS) outbreaks have also caused death and disability [1,2].^1^,^2^,^3^,^4^,^5^

The SARS coronavirus 2 (SARS-CoV-2) pandemic, which emerged in China at the end of 2019 and spread around the world very quickly, was declared the coronavirus disease 2019 (COVID-19) pandemic by the World Health Organization (WHO) on March 11th, 2020, and caused 5.4 million deaths (December 2022) in approximately 3 years.

**Figure 1:**
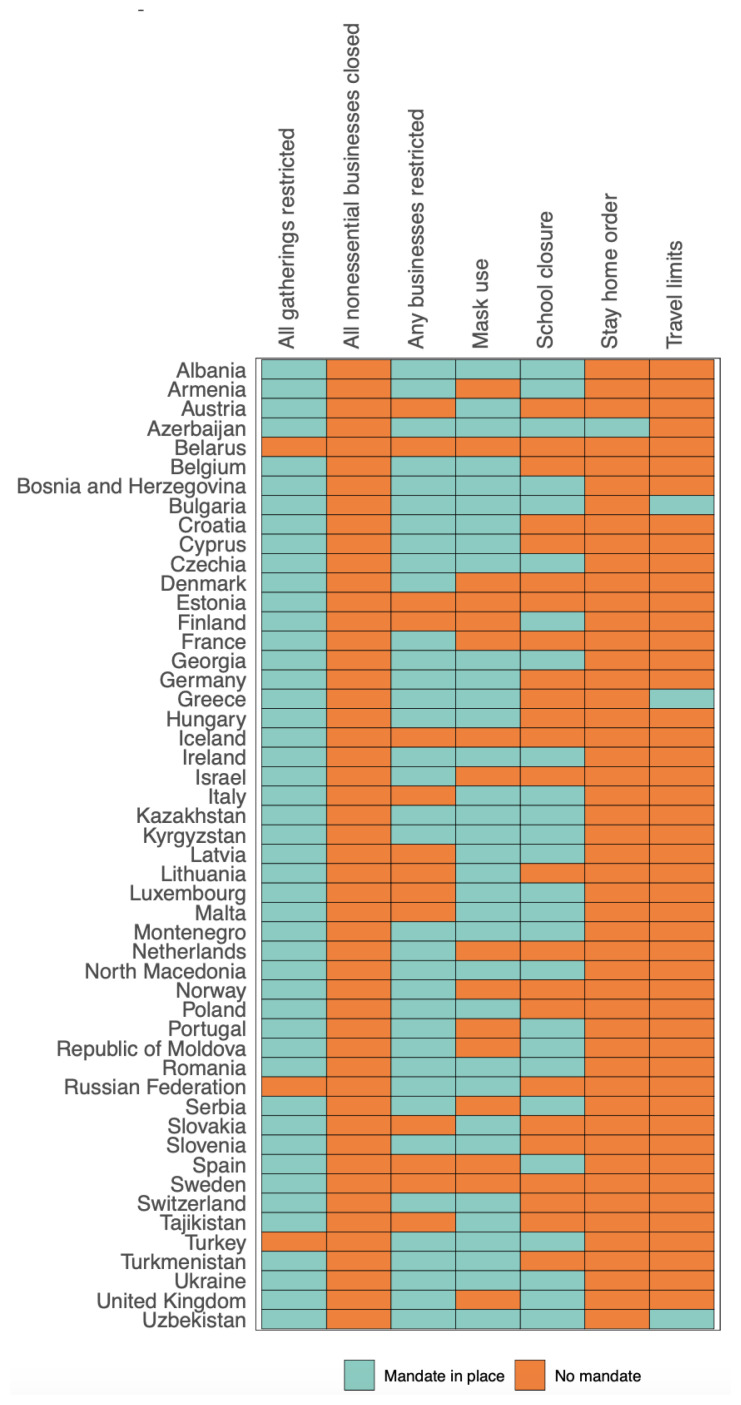
National enforcement practices in countries of the WHO European region, September 2020 (some businesses restricted, some meetings restricted, mandatory use of face masks, school closures, stay-at-home orders, travel restrictions).

Since the beginning of the COVID-19 pandemic, the WHO, the Centers for Disease Prevention and Prevention (CDC) and the ministries of health of the countries have published information guides, posters, brochures, and infographics on protection from the COVID-19 virus. All countries have developed various strategies to mitigate the effects of the pandemic by increasing the capacity of their health infrastructure. People were constantly reminded about wearing face masks, social distancing, and cleanliness rules by both authorities and the media. Domestic and international travel restrictions were implemented in many countries, and measures against transmission routes with a few minor differences were implemented through legal regulations in all countries dealing with the pandemic (Figure 1).

During the pandemic, the Republic of Türkiye Ministry of Health established filiation teams and telephone counseling centers for the detection and follow-up of and communication with COVID-19-positive patients and their contacts and provided 24/7 services. Positive patients followed-up with filiation services were referred to hospitals before the development of severe clinical pictures and it was attempted to prevent COVID-19-related complications. During the pandemic, the diagnosis, treatment, follow-up, and processes of all patients and their contacts were monitored with a patient tracking system developed by the Republic of Türkiye Ministry of Health within the Public Health Management System (HSYS). With the Filiation and Isolation Tracking System (FITAS) and Spatial Business Intelligence (MIZ) systems, it was ensured that filiation activities in the field were carried out faster [3].^1^,^2^,^3^,^4^

There is no doubt that efforts to combat the COVID-19 pandemic led to significant side gains in many areas. Since sensitivity to comply with general hygiene rules increased during the COVID-19 pandemic, and contact between people was interrupted by wearing face masks and physical distancing, it is thought that these health behaviors have led to an important increase in the protection of many infectious diseases, especially respiratory infections, and may have important contributions. In this study, it was aimed to evaluate the positive effects of health behaviors (general hygiene, wearing face masks, physical distancing, and travel restrictions) that increased during the COVID-19 pandemic in Ankara Province, Türkiye, on the prevention of other infectious diseases.

Included herein were acute intestinal tract infections with International Classification of Diseases, Tenth Revision (ICD-10) diagnostic codes A09 (diarrhea and gastroenteritis presumed to be of infectious origin), R11 (nausea and vomiting), and K52 (other noninfectious gastroenteritis and colitis), as well as influenza, tuberculosis (TB), measles, chickenpox, malaria, and meningococcal meningitis.

### 1.1. Acute intestinal infection (AII)

AII is one of the most common seasonal infectious diseases in many countries around the world and continues to be a serious public health problem. According to a report by The European Food Safety Authority, AII cases are reported to be present throughout the year, but some months are associated with higher incidences.

T.C. The Ministry of Health has established the Infectious Disease Surveillance and Early Warning System (IZCI) in order to monitor all studies (filiation, case investigation, contact tracing, etc.), reporting for these studies, and signal and analysis results for the early detection of health threats in an electronic environment. Data with ICD-10 diagnosis codes A09 (diarrhea and gastroenteritis of presumed infectious origin), R11 (nausea and vomiting), and K52 (other noninfectious gastroenteritis and colitis), defined as AIIs, are monitored with the IZCI system. When a C4 increase signal is detected in the IZCI system, the patient lists in the system are examined to assess whether there is a significant clustering of cases in terms of age groups and addresses, and surveillance and intervention studies are carried out in order to prevent the spread of ACI [4–7].^5^,^6^

### 1.2. Influenza (flu)

Influenza surveillance studies are carried out sensitively all over the world to monitor the activity of the virus and detect and control outbreaks in advance. Due to these studies, influenza is closely and safely monitored. The Global Influenza Surveillance and Response System (GISRS) conducts global influenza surveillance activities worldwide in collaboration with 143 national influenza surveillance centers in 129 countries and the WHO. Data collected by regions and countries include those from respiratory specimens systematically collected for polymerase chain reaction testing from a representative number of primary care physicians from patients with influenza-like illness or acute respiratory infection (ARI). Countries report weekly epidemiological and virological influenza data to the European Surveillance System (TESSy) hosted at the European Centre for Disease Prevention and Control (ECDC). The time interval for the influenza season is between week 40 and week 20 of the following year.^7^,^8^ In Türkiye, sentinel surveillance studies are carried out with volunteer family physicians working in 220 Family Health Centers in 21 provinces. In this way, data are obtained in real time and effectively.

The data obtained within the scope of surveillance are evaluated and published as a weekly report at www.grip.gov.tr by the General Directorate of Public Health.^9^ Within the scope of preventing the transmission and spread of the disease in the community are regulations such as avoiding closed and crowded areas as much as possible, wearing medical face masks in these areas, and complying with personal hygiene rules, especially hand hygiene [8,9].

### 1.3. TB

TB is one of the top 10 leading causes of death from infection, occurring in approximately 10 million people worldwide each year. The WHO reported 6.4 million officially registered new TB cases worldwide in 2017, which represent 64% of the 10 million new cases expected for 2017. The most effective methods for prevention are vaccination, avoiding contact with infected people, and wearing face masks [10–13].

### 1.4. Measles

The Measles Elimination Program adopted in the WHO European Region, which aims to eliminate measles and rubella, has been implemented since 2002. Türkiye is at risk of importation-related infection due to its geographical location and increasing population movements in recent years. Imported measles cases come to Türkiye from other countries and unvaccinated or under-vaccinated individuals from various age groups can be affected by these patients and become ill. Due to outbreaks in various countries in the WHO European Region (Ukraine, Romania, Bulgaria, Greece, Serbia, Israel, Italy, France, Germany, Georgia, Albania, and Russia) and Syria since 2005, Türkiye has been experiencing infection related to imported cases since June 2012. The most effective methods for protection are vaccination, avoiding contact with sick people, and wearing face masks.^10^

### 1.5. Chickenpox

Chickenpox is a highly contagious disease characterized by widespread, itchy, fluid-filled rashes, often accompanied by fever, usually in early childhood. The most effective methods for protection are vaccination, avoiding contact with sick people, and wearing face masks.^13^

### 1.6. Malaria

Malaria is a widespread infectious disease in 87 countries around the world, mostly in Africa. The WHO has reported a decline in the number of malaria cases and deaths worldwide since 2000.

According to the WHO World Malaria Report, globally, the estimated 238 million malaria cases in 2000 decreased to 229 million in 2019, and malaria-related deaths steadily decreased from 736 thousand in 2000 to 409 thousand in 2019. Travelers to high-risk areas should take the recommended precautions [14,15].

### 1.7. Meningococcal meningitis

Invasive meningococcal disease serogroups A, B, C, W135, X, and Y are responsible for 13 serogroups and their distribution varies worldwide. The most common and dangerous diseases caused by *Neisseria meningitidis* are meningococcemia and meningitis.

The main reservoir of meningococcal infections is humans. Meningococcal meningitis is transmitted by direct contact or droplet transmission from the nose or throat of an infected person.^11^ The most effective methods for protection are vaccination for those traveling to high-risk areas, avoiding contact with sick people, and wearing face masks [16].

## 2. Materials and methods

This study was designed retrospectively. The data of foodborne diseases (acute intestinal tract infections), respiratory diseases (influenza, TB, measles, varicella, chickenpox), and travel-related diseases (malaria, meningitis), selected for their close relation to the general hygiene rules in the last 2 years before the pandemic (prepandemic period; January 2018–December 2019) in Ankara Province, were evaluated by comparing them with the data of the 2-year period of the pandemic (pandemic period; January 2020–December 2021). Ankara Province was taken as a sample for this comparison.

Research data were obtained from the IZCI system, sentinel influenza surveillance studies, Turkish National Tuberculosis Surveillance Survey (TUTSA), Basic Health Statistics Module (TSİM/VSD17), National Tuberculosis Surveillance (UTS), and the Public Health Management System (HSYS) modules. Each disease included in the scope of this research has legislation and field guidelines prepared by the Ministry of Health.

This study is expected to make a scientific contribution to the literature in terms of emphasizing the protective effect of the measures implemented during the pandemic against selected infectious diseases.

This study was limited to acute intestinal tract infections, influenza, TB, measles, varicella, malaria, and meningococcal meningitis. Undoubtedly, the measures taken during the pandemic period have also had an impact (either decreasing or increasing) on other diseases. There are other examples in the pandemic literature. The study herein compared the prepandemic period with the pandemic period using data from the retrospective surveillance records of selected infectious disease cases.

An application was made to the General Directorate of Health Services of the Turkish Ministry on November 16th, 2020, with application form 2020_11_16T11_53_56 on November 16th, 2020, for the research permit of the doctoral thesis titled “Positive effects of health behaviors acquired during the COVID-19 pandemic process on the prevention of other infectious diseases” and the study was accepted by the Scientific Research Platform, in a letter dated November 17th, 2020.

In addition, the Ankara Public Health Services Directorate of the Ankara Primary Health Services Research Requests Evaluation Commission granted research permission in a letter dated December 2nd, 2020 and numbered 129998426. Permission was granted by the Ankara Provincial Health Directorate for publication as an article in a scientific journal in a letter dated December 1st, 2021 and numbered 153194792.

The data were analyzed after being checked. The number of cases were expressed as frequencies, and the 1-sample chi-squared test (chi square calculator for goodness of fit) was used in the statistical analysis and the statistical significance level was accepted as 0.05.

## 3. Results

### 3.1. Diseases whose transmission has decreased due to general hygiene rules

In Türkiye, AII cases are monitored, and early intervention is carried out through the IZCI digital program.

In Ankara Province, there was a relative increase in the number of AII cases due to the high temperatures experienced between August and September due to foods kept outside (home, restaurant, mass meal ceremonies, etc.).

However, during the pandemic process, a statistically significant difference was found when the prepandemic period and pandemic period data with ICD-10 diagnosis codes A09, R11, and K52, defined in the IZCI system, were compared (X^2^ = 224508.368, p < 0.001).

In particular, it was found that cases coded A09 decreased by −45.49% in the pandemic period compared to the prepandemic period and there was a statistically significant difference (X^2^ = 25083.583, p < 0.001) (Table).

**Figure 2:**
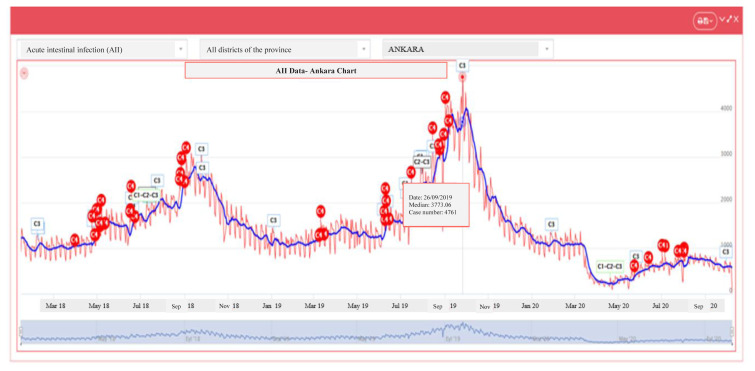
Distribution of AII cases by month in the prepandemic period (2018–2019).

**Figure 3:**
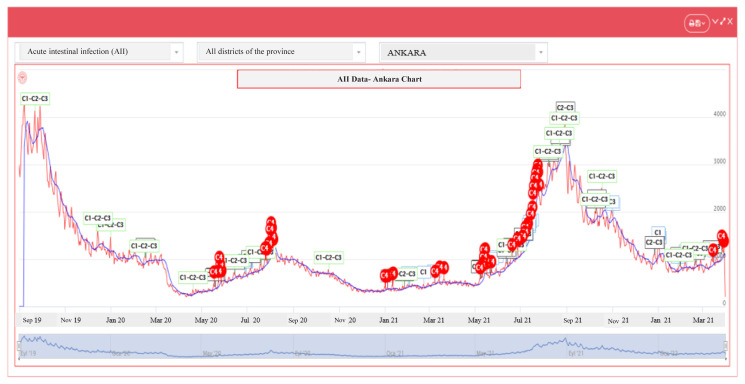
Distribution of the AII cases by month in the pandemic period (2020–2021).

The IZCI graphs show a significant decrease in the AII cases in 2020–2021, which were high between August and October 2018–2019 (Figures 2 and 3).

### 3.2. Diseases whose transmission has decreased due to wearing face masks and physical distancing

The number of TB, measles, and chickenpox cases has decreased rapidly since March 2020 due to wearing face masks, school closures, and quarantine measures taken as a result of COVID-19. When the prepandemic period and pandemic period were compared, a statistically significant difference was found in the number of TB, measles and varicella cases. In particular, measles cases decreased by −95.05% in the pandemic period compared to the prepandemic period and there was a statistically significant difference (X^2^ = 86.943, p < 0.001) (Table).

In its report dated August 11th, 2020, the WHO Global Influenza Program stated that the high increase in demand for COVID-19 testing strained laboratory capacity in many countries and significant disruptions were experienced in influenza surveillance, the weekly reporting of data was delayed, and influenza activity was low in countries that could test and report. The same problem was observed in Ankara Province; therefore, the number of influenza cases reported to the system was included in the study.

### 3.3. Diseases whose transmission has decreased due to travel restrictions

After March 27th, 2020, imported malaria cases decreased following the complete suspension of international flights. Due to the travel ban, more than 100 thousand Turkish citizens who were stranded outside of Türkiye (in 142 countries) as civil servants, students, workers, or tourists were given permission to return to Türkiye by the Ministry of Foreign Affairs of the Republic of Türkiye. The majority of the imported cases seen in Ankara Province in 2020 were Turkish citizens who resided abroad and returned to Türkiye. The cases seen after the opening of international flights in 2021 were foreign nationals. It was found that malaria cases decreased by 60.87% in the pandemic period compared to the prepandemic period and there was a statistically significant difference (X^2^ = 20.381, p < 0.001) (Table).

Studies have shown that the complete suspension of international flights, closure of borders between countries, hygiene measures, quarantines, and school closures during the pandemic led to a dramatic decrease in meningococcal meningitis cases. In the current study, a significant decrease in the number of cases was also observed (14 cases in 2018–2019 and only 4 cases in 2020–2021) (Table).

## 4. Discussion

In the current study, it was determined that there was a significant decrease in AII cases with ICD 10 A09, R11, K52 codes in 2020–2021, which were high between August and October 2018–2019. Especially in cases coded A09, a statistically significant decrease was observed when the prepandemic period and pandemic period were compared.

In a study conducted by Karamustafa et al. [17] in Türkiye, it was reported that establishments performed general disinfection procedures more meticulously. Some changes, such as ventilation of the environment at certain intervals during service, temperature control, use of disposable sauce sets, spaced table layout, use of QR-coded menus, and service on stretch plates are noteworthy.

A study conducted in the UK by Ondrikova et al. [18] focused on norovirus and campylobacter, as they are the most common viral and bacterial causative agents of infectious intestinal disease (IID) in the UK and found that the overall decline for norovirus in 2020 was 47%–79%, while for campylobacter it varied over time, for example 19%–33% in April and 7% in August. A decrease in norovirus transmission and incidence was thought to be due to behavioral changes such as increased hand washing, social distancing, and personal hygiene, and infection control measures implemented to prevent COVID-19.

Tanislav et al. [19] conducted a study in Germany, and reported a significant decrease in gastrointestinal infections (GIIs) (40%, p < 0.001) in family physician practices, suggesting that a gradual increase in hygiene awareness is very important to prevent the spread of COVID-19 and that these measures may have also affected the spread of other infectious diseases, such as respiratory tract infections and GIIs.

In a study conducted by Angoulvant et al. [20] in France, pediatric emergency admissions to hospitals were examined to measure the impact of school closures and national quarantine on viral and nonviral infections at the onset of the COVID-19 pandemic. It was reported that outpatient admissions decreased by 68%, hospitalizations decreased by 45%, and acute gastroenteritis, the common cold, bronchiolitis, and acute otitis media significantly decreased by over 70% compared to the expected values.

These studies were consistent with the current study in terms of showing a decrease in the disease group of AIIs.

In the current study, when the number of samples taken within the scope of sentinel surveillance in influenza cases in the prepandemic period and pandemic period were compared, no statistically significant difference was found.

In its report dated August 11th, 2020, the WHO Global Influenza Program stated that the high increase in demand for COVID-19 testing has strained laboratory capacity in many countries and that there have been significant disruptions in influenza surveillance, weekly reporting of data has been delayed, and influenza activity is low in countries that can test and report it.

In the present study, a statistically significant difference was found when the number of patients with TB treated in the prepandemic period and pandemic period was compared.

In a study conducted by Lai et al. [21] in Taiwan, it was reported that there was a significant decrease in TB cases in 2020 compared to previous years, there was a difference in the slope between each of them, and no such downward trend was observed in human immunodeficiency virus and hepatitis C virus cases in the same period, unlike TB. They underlined that the decline in the TB trend can be explained by the measures and interventions taken to control SARS-CoV-2 transmission since the beginning of 2020, as a result, they noticed the significant decrease in TB activity and underlined that success can be achieved in aerosol prevention with droplet preventive measures.

Komiya et al. [22] determined that the number of newly diagnosed TB patients during the COVID-19 outbreak in Japan, as seen in Taiwan, decreased significantly, especially since March 2020, but in contrast, the detection rate of TB decreased due to the COVID-19 outbreak. Indeed, especially during quarantine periods, medical checks were delayed due to limited access to some clinics in hospitals, as in many regions, and it was thought that people may not have presented with nonurgent symptoms. They argued that such a situation may have deprived physicians of the opportunity to suspect and test for TB infection.

In a multicenter study conducted by Driessche et al. [23] in South Africa and Belgium, they emphasized that wearing face masks in the COVID-19 pandemic could be a silver lining (opportunity-every bad situation has a positive side to it) for the TB public health response, that wearing face masks is important in the fight against the TB pandemic, especially in patients with TB, and that wearing face masks should be widely accepted for TB control.

These studies, including the contribution of wearing face masks to the decrease in the number of TB cases, were found to be compatible with the current study.

In the present study, a statistically significant difference was found when comparing the number of measles cases in the prepandemic period and pandemic period.

In their study in Guangzhou, China, Wu et al. [24] investigated the effect of COVID-19 control measures on measles, rubella, varicella, and herpes zoster cases. Data on measles, rubella, varicella, chickenpox, herpes zoster, influenza, and pneumonia from the Chinese Disease Prevention and Control Information System for a total of 4 years, 2020 and the previous 3 years, were analyzed to determine the impact of control measures taken during the pandemic, such as wearing face masks, social distancing, body temperature measurement, quarantines, and the closure of schools, shopping malls, and other places, on other infectious respiratory diseases. It was found that varicella cases decreased significantly compared to previous years, only 63.96% of rubella cases were reported only in 2019, only 4 measles cases were reported in 2020, and it was emphasized that influenza and pneumonia cases both decreased significantly during the pandemic period.

During the COVID-19 pandemic, control measures such as quarantine and the closure of schools, shopping malls, and other places, as well as wearing face masks, social distancing, and hygiene measures have been observed in studies to lead to a sharp decline in measles cases, especially in 2020, which is consistent with the current study.

Since March 2020, the decline seen in measles cases has also been observed in chickenpox cases. There was a dramatic decline in varicella cases due to wearing face masks, school closures and quarantine measures.

In the present study, a statistically significant difference was found when the number of varicella cases in the prepandemic period and pandemic period were compared.

In a study conducted by Yiğit and Parlakay in Ankara at Bilkent City Hospital [25], They reported an 85.6% decrease in bronchiolitis cases during the pandemic period compared to previous years, the chickenpox peaks seen in previous years were not seen, and measures that restricted social life and prioritized social distancing such as wearing face masks, quarantines, school closures, hand hygiene, and travel restrictions prevented the spread of viral infections. However, although there was a decrease in childhood infections due to the preventive measures taken during the pandemic period, they drew attention to the need for sensitivity to prevent disruption in the implementation of childhood vaccines, which is the most important of the measures to prevent reemergence in the coming years.

During the COVID-19 pandemic, it was observed in studies that the varicella peaks seen in previous years were not observed due to quarantines and school closures, which is consistent with the results herein.

In the current study, a statistically significant difference was found when the number of malaria cases was compared in the prepandemic period and pandemic period.

Guo et al. [26] conducted a study in China, in which the impact of control strategies on vector-borne diseases was investigated. When data on dengue fever, epidemic encephalitis B, typhus, malaria, and leishmaniasis were compared between 2015 and 2019, and 2020, there was an 89.93% decrease in dengue incidence and 61.89% decrease in malaria incidence.

In their study conducted in Switzerland, Steffen et al. [27] emphasized that quarantine and travel restrictions significantly reduced the incidence of vector-borne diseases. During the quarantine period, nonendemic imported malaria cases reported in 2020 decreased by 86.1%.

Ullrich et al. [28] performed their study in Germany and reported that respiratory disease rates decreased by 86% for measles and 12% for TB, gastrointestinal disease rates decreased by 83% for rotavirus and 7% for yersiniosis, and imported vector-borne disease rates decreased by 75% for dengue fever, 73% for malaria, and only 58% for tick-borne encephalitis.

It was observed in studies that measures such as the complete suspension of international flights and the closure of borders between countries during the COVID-19 pandemic led to a decrease in vector-borne cases, especially malaria, and especially in countries other than those in Africa, which is consistent with the results herein.

In addition to travel restrictions, mandatory conjugated measles-mumps (MM) vaccination of travelers to MM endemic countries has a high impact on the decrease in MM cases. These studies, which included the contribution of travel restrictions to the reduction in the number of cases, were found to be consistent with the current study.

When the data of the diseases included in the present study were evaluated, it was understood that the dramatic decrease seen during the pandemic period was also observed in many studies conducted in the literature. It cannot be denied that increased sensitivity to general hygiene rules, wearing face masks, social distancing, and travel restrictions during the COVID-19 pandemic provided significant gains in protection from many infectious diseases.

## 5. Conclusions

One of the biggest threats to global health is uncontrolled pandemics due to highly pathogenic microorganisms. The COVID-19 pandemic has emerged as a unique global threat to all of humanity. The health systems of even the most developed countries have been shaken by the pandemic, with economic crises and challenges in all walks of life.

However, there is no doubt that the studies carried out and the behaviors gained during the fight against the pandemic have provided very important gains in many areas. Undoubtedly, the habits that the pandemic period has brought to society, especially the awareness of protection from infectious diseases, will guide us in protecting our future health.

In our future lives, we will talk about before and after the pandemic. We will discuss what the new normal in our lives will be for the days to come. It is predicted that health literacy, which has developed with the information gained and measures taken during the COVID-19 pandemic, will show its positive effects in protecting us against not only COVID-19, but also many infections, especially respiratory infections, and that it will be possible to carry this gain further with sustainable measures in the ongoing process. With the awareness that living in crowded environments and as a highly mobile population, that unhygienic habits are unfavorable for the spread of all infectious diseases, care must be taken to constantly apply the precautions in relation to healthy living in our new lifestyle.

Most importantly, today, when transportation is so fast and easy, infectious diseases have the potential to easily cross borders. Around the world, with 3200 airports, 60 thousand routes, and 100 thousand daily flights, 6 million people fly from one place to another every day.

With the increase in international travel, the importance of travel health interventions is increasing due to infectious diseases, unhygienic conditions, and exposure to vectors in destination countries. In order to prevent the spread of infectious diseases in the global village (world) we live in, their emergence should be predicted, detected, and intervened quickly and effectively. In order to manage possible outbreaks, countries should always stay up-to-date in terms of taking precautions, being prepared, early detection, and rapid response.

## Figures and Tables

**Table:** Effect of health behaviors acquired during the COVID-19 pandemic on some infectious diseases, statistical analysis of cases in prepandemic period (2018–2019) and pandemic period (2020–2021).

Diseases with declining transmission	Total number of cases in 2018–2019	Total number of cases in 2020–2021	Percentage change	X^2^	p-value
With general hygiene					
AIIs	1,353,108	677,854	−49.91	224508.368	<0.001
A09	187,336	102,126	−45.49	25083.583	<0.001
R11	624,798	372,192	−40.44	64002.439	<0.001
K52	540,674	203,716	−62.33	124546.548	<0.001
With face masks and social distancing					
Influenza	2821	2936*	*	2.257	<0.05
TB	1269	1023	−19.39	26,403	<0.001
Measles	101	5	−95.05	86.943	<0.001
Chickenpox	2451	458	−81.32	1366.805	<0.001
With travel restrictions					
Malaria	69	27	−60.87	20.381	<0.001
M. Meningitis	14	4	−71.43	5.556	<0.05

* Number of cases recorded with the surveillance system with an influenza diagnosis.

## Footnotes

1 6”https://www.healthdata.org/sites/default/files/files/Projects/COVID/briefing_EURO_092420.pdf”https://www.healthdata.org/sites/default/files/files/Projects/COVID/briefing_EURO_092420

2 .pdf [accessed 28 May 2022]. Republic of Türkiye Ministry of Health (2022). HES [online]. Website https://hayatevesigar.saglik.gov.tr/hes-eng.html [accessed 11 June

3 2022]. Republic of Türkiye Ministry of Interior (2020). Circular exempting people aged 65 and over/under 20/chronically ill from curfew restrictions [online]. Website https://www.icisleri.gov.tr/81-il-valiligine-sehir-giris---cikis-tedbirler

4 i-konulu-yeni-bir-genelge [accessed 11 June 2022]. 9ID-19 posters for the public [online]. Website https://covid19.saglik.gov.tr/TR-66259/halka-yonelik.html [accessed 11 June 2022].

5 Republic of Türkiye Ministry of Health, General Directorate of Public Health (2019). IZCI system [online]. Website https://covid19.saglik.gov.tr/TR-66259/halka-yonelik.html [accessed 26 September 2019 and 01 April 2021].

6 Republic of Türkiye Ministry of Health (2015). Bulaşıcı Hastalıklar Genelgesi [online]. Website https://hsgm.saglik.gov.tr/depo/Mevzuat/Genelgeler/2015-18_Bulasici_Hastaliklarin_Ihbar_ve_Bildirim_Sistemi_Genelgesi.pdf [accessed 11 June 2022].

7 World Health Organization (2020). Maintaining surveillance of influenza and monitoring SARS-CoV-2, adapting global influenza surveillance and response system (GISRS) and sentinel systems during the COVID-19 pandemic [online]. Website https://www.who.int/publications/i/item/maintaining-surveillance-of-influenza-and-monitoring-sars-cov-2-adapting-global-influenza-surveillance-and-response-system-(gisrs)-and-sentinel-systems-during-the-covid-19-pandemic [accessed 2 July 2022].

8 World Health Organization (2011). Global Influenza Surveillance Network Manual for the laboratory diagnosis and virological surveillance of influenza [online]. Website https://www.who.int/publications/i/item/manual-for-the-laboratory-diagnosis-and-virological-surveillance-of-influenza [accessed 2 July 2022].

9 Republic of Türkiye Ministry of Health (2022). 2017-2018 weekly influenza reports [online]. Website https://grip.gov.tr/tr/haftalik-influenza-raporu [accessed 2 July 2022].

10 Republic of Türkiye Ministry of Health (2010). Circular on measles/rubella and congenital rubella syndrome surveillance, dated 2010 April 01 and numbered 18617-2010/20 [online]. Website https://www.saglik.gov.tr/TR,11146/kizamik-kizamikcik-ve-konjenital-kizamikcik-kks-surveyansi-genelgesi-2010.html [accessed 2 July 2022].

11 Republic of Türkiye Ministry of Health (2022). Menenjit [online]. Website https://hsgm.saglik.gov.tr/tr/hastaliklar/menenjit.html [accessed 11 July 2022].

**Funding:** The authors have no relevant financial or nonfinancial interests to disclose.
